# Morpho-Syntactic Abilities of Unbalanced Bilingual Children: A Closer Look at the Weaker Language

**DOI:** 10.3389/fpsyg.2018.01318

**Published:** 2018-08-13

**Authors:** Natalia Meir

**Affiliations:** ^1^Department of English Literature and Linguistics, Bar Ilan University, Ramat Gan, Israel; ^2^Department of Communication Sciences and Disorders, University of Haifa, Haifa, Israel

**Keywords:** morpho-syntax, unbalanced language development, the Weaker Language, delay, deviance, Specific Language Impairment (SLI)

## Abstract

Previous studies evaluating morpho-syntactic abilities in the Weaker Language of unbalanced bilingual children are scarce; and they bring inconclusive evidence on the nature of the Weaker Language development. The current study looked into morpho-syntactic profiles of bilingual Russian–Hebrew speaking children in the Weaker Language [the Weaker Heritage Language (HL-Russian) and the Weaker Societal Language (SL-Hebrew)] as compared to balanced bilinguals, unbalanced bilinguals in the Dominant Language and bilinguals with Specific Language Impairment (SLI). Four groups of bilingual children aged 5;5–6;5 participated: unbalanced bilinguals with the Weaker HL-Russian and the Dominant SL-Hebrew (HL-weak: *n* = 39), unbalanced bilinguals with the Weaker SL-Hebrew and the Dominant HL-Russian (SL-weak: *n* = 19); balanced bilinguals (BB: *n* = 38), and bilinguals with SLI (biSLI: *n* = 23). Children’s morpho-syntactic abilities in both languages were investigated using LITMUS (Language Impairment Testing in Multilingual Settings) Sentence Repetition Tasks (based on Marinis and Armon-Lotem, 2015). Quantitative analysis of morpho-syntactic abilities showed that unbalanced bilinguals scored lower in the Weaker Language as compared to balanced bilinguals and unbalanced bilinguals in the Dominant Language, yet, higher than bilinguals with SLI. Error patterns were similar across bilingual groups with TLD and could be traced to cross-linguistic influence. By contrast, error profiles of unbalanced bilinguals in the Weaker Language and bilinguals with SLI bore fundamental differences. Whereas unbalanced bilinguals in the Weaker Language opted for complex structures, relying on the available resources from the Dominant Language; bilinguals with SLI simplified complex syntactic structures. To conclude, the study shows that the Weaker Language of unbalanced bilinguals with TLD develop qualitatively similarly to the languages of balanced bilinguals and the Dominant Language in unbalanced bilinguals, albeit delayed or influenced by the Dominant Language to a larger extent. Conversely, the study brings evidence that linguistic profiles of unbalanced bilinguals with TLD in the Weaker Language and bilinguals with SLI differ, pointing at a deviant pattern of acquisition in children with SLI.

## Introduction

Linguistic abilities of bilingual children with typical language development (TLD) are unevenly distributed within and across the two languages (Kohnert, 2010). Typically, most bilingual children have one language which is more dominant (i.e., stronger, more preferred) than the other language. This has been noted for simultaneous bilingual children (those bilinguals who are exposed to both of their languages early in childhood) and sequential bilingual children [those who first acquire the Heritage Language (HL) and then are exposed to the Societal Language (SL)] (e.g., Pearson et al., 1993; Schlyter, 1994; Müller and Kupisch, 2003; Bernardini and Schlyter, 2004). Language dominance as well as language preference changes over the life span of bilinguals (e.g., De Houwer, 1990; Montrul, 2008; Gathercole and Thomas, 2009; Polinsky, 2018). For example, in sequential bilingual acquisition, the SL usually starts as the Weaker Language and often becomes the Dominant Language over time. Conversely, the HL starts as the Dominant language and often becomes the Weaker Language as the SL gains dominance. Previous studies report contradicting findings on the nature of the Weaker Language development. Some studies suggest that unbalanced bilinguals in the Weaker Language show similar trajectories to the ones observed in monolingual children, balanced bilinguals (BB) and unbalanced bilinguals in the Dominant Language, yet this pattern is delayed (e.g., Müller and Kupisch, 2003; Bernardini and Schlyter, 2004; Antonova Ünlü and Li, 2016, 2017, 2018). Conversely, some studies show that the Weaker Language development does not follow the monolingual trajectory, i.e., it resembles adult L2 acquisition or it is influenced by the Dominant Language. Numerous studies have shown that morpho-syntactic abilities of bilingual children are susceptible to cross-linguistic influence and bilinguals diverge from monolingual baseline grammars (Müller and Hulk, 2001; Paradis and Navarro, 2003; Argyri and Sorace, 2007; Kupisch, 2007; Meir et al., 2017; Sorace and Serratrice, 2009). The current study does not aim to compare bilingual children to monolingual “golden” standards, rather it is devised to investigate different types of bilingual language development: balanced versus unbalanced, typical versus atypical. These patterns of bilingual language development are investigated in Russian–Hebrew speaking bilingual children. Russian–Hebrew bilingualism offers a unique opportunity to test cross-linguistic influence since some morpho-syntactic properties are configured similarly in both languages (e.g., verbal inflections), while some properties vary across the two languages (e.g., case morphology, aspectual marking, definiteness).

The goal of the current study is twofold. First, it aims to fill the gap created by “the weak interest in the Weaker Language” (Bernardini, 2017). The study investigates morpho-syntactic skills of two groups of unbalanced bilinguals: bilinguals with the Weaker Heritage Language (HL-Russian) and the Weaker Societal Language (SL-Hebrew). Second, the study aims to add to the delay-versus-deviance debate by comparing linguistic profiles of unbalanced bilingual children with TLD and bilingual children with Specific Language Impairment (SLI). This comparison is intended to unravel the underlying nature of grammatical representations in the two populations.

To evaluate morpho-syntactic abilities, children’s performance on LITMUS (Language Impairment Testing in Multilingual Settings) Sentence Repetition Tasks (based on Marinis and Armon-Lotem, 2015) were administered in both languages of bilingual children (HL-Russian and SL-Hebrew). Sentence Repetition tasks are widely used to assess morpho-syntactic abilities of monolingual and bilingual children. Sentence Repetition tasks have been shown to be highly effective in discriminating children with typical and atypical language development in monolingual and bilingual populations (e.g., Conti-Ramsden et al., 2001; Archibald and Joanisse, 2009; Klem et al., 2015; Meir et al., 2016; Antonijevic et al., 2017; Gavarró, 2017; Hamann and Abed Ibrahim, 2017; Theodorou et al., 2017; Fleckstein et al., 2018; among many others). In the following subsections, quantitative and qualitative characteristics of the Weaker Language of unbalanced bilinguals are discussed as compared to BB and unbalanced bilinguals in the Dominant Language. Second, the trajectory of the Weaker Language development in unbalanced bilinguals with TLD is discussed in terms of delay and deviance. Third, studies on atypical language development in children with SLI are reviewed. Finally, specific research questions and predictions for the current study are presented.

### The Weaker Language vs. the Dominant Language: Quantitative Characteristics

Determining the Weaker and the Dominant Language in bilinguals is not an easy task and it poses great challenges to linguists, educators and speech pathologists. Meisel (2007) defines a language as “weak” or “non-dominant” based on input and output characteristics, on the one hand, and language skills, on the other hand. It is suggested that the Weaker Language is (a) rarely actively used, (b) the other language is strongly preferred over an extended period of time, and (c) the development of the Weaker Language is less advanced than that of the other language(s).

Previous studies rely on quantitative differences between the two languages of a bilingual child. Quantitative discrepancies in scores across the two languages are viewed as a token of unbalanced bilingual language development. For example, many studies, especially those on younger bilinguals, use mean length of utterance (MLU) and directionality of code-mixing as indices of language dominance (e.g., Schlyter, 1994; Jisa, 2000; Bernardini and Schlyter, 2004). Some studies determine language dominance based on direct language proficiency scores (e.g., Paradis et al., 2003; Iluz-Cohen and Armon-Lotem, 2013). Children who obtain higher scores in one language and lower scores in the other language are labeled as unbalanced bilinguals, higher scores in the language signify the Dominant Language, while lower scores are viewed as a sign of the Weaker Language. Other studies rely on quantitative differences in exposure and output characteristics (e.g., Thordardottir, 2011; Hoff et al., 2012; Gathercole et al., 2014; Unsworth, 2016; de Almeida et al., 2017). For example, Hoff et al. (2012) used estimates of exposure at home to determine dominance of Spanish–English toddlers. Children who had above 70% of exposure to the language at home, were labeled as dominant in that language. In the study of de Almeida et al. (2017) on bilinguals with SL-French, several measures were used to compute a dominance score: exposure in each of the child’s languages, Age of Onset of bilingualism (AoO), frequency of early exposure, diversity of early contexts of exposure, Length of Exposure (LoE), present use of each language at home, present use during different activities and with friends, and number of years the child has spent in elementary school. Some studies combine direct and indirect indices of language dominance, i.e., look at the discrepancies in the proficiency scores and discrepancies in parental ratings of children’s language proficiency (e.g., Gutiérrez-Clellen et al., 2006; Bedore et al., 2011).

As demonstrated above, different measures are used in the literature to determine language dominance in bilingual children. Different indices might result in different classification labels for bilinguals. This has been demonstrated by Bedore et al. (2012) which compared indices of children’s input and output characteristics, based on parental questionnaires, as well as language skills in HL-Spanish and SL-English among 1029 Spanish–English bilinguals with different levels of language dominance: functional monolingual English, bilingual English-Dominant, BB, bilingual Spanish-Dominant and functional monolingual Spanish. The child’s current language use was found to be the stronger predictor of the children language performance as measured by direct assessment of language skills in HL and SL. Lust et al. (2016) showed a discrepancy between difference indices of language exposure for Korean–English bilinguals suggesting that parental reports should be supplemented by direct measures of language assessment.

To sum up, quantitative discrepancies across different measures (e.g., MLU, directionality of code-mixing, parental ratings, exposure patterns, language scores (vocabulary and morpho-syntax) are used to determine language dominance in bilinguals. Yet, these quantitative measures do not shed light on qualitative characteristics of the Weaker Language. The next subsection will discuss qualitative properties of the Weaker Language of unbalanced bilinguals and trajectories of the Weaker Language development with the main focus on morpho-syntax.

### Morpho-Syntactic Abilities in the Weaker Language of Bilinguals With TLD: Delayed or Deviant

The Weaker and the Dominant Languages of a bilingual child vary quantitatively as it has been demonstrated in the previous section. Yet, with respect to the qualitative differences there is no agreement. On the one hand, linguistic profiles of unbalanced bilinguals in the Weaker Language resemble those of BB and bilinguals in the Dominant Language. Some studies even show that the Weaker Language development is qualitatively similar to the one of monolinguals. Conversely, some studies show that error patterns in the Weaker Language differ from those of monolinguals, BB and bilinguals in the Dominant Language. This gave rise to two competing hypotheses on the nature of the Weaker Language development: the Delay Hypothesis and the Deviance Hypothesis.

Similarities in error profiles of unbalanced bilinguals in the Weaker Language and other groups of children (e.g., monolinguals, BB and unbalanced bilinguals in the Dominant Language) provide support for the Delay Hypothesis. For example, Müller and Kupisch (2003) showed that despite quantitative differences in the development of the Weaker Languages of French–German unbalanced bilinguals, the Weaker and the Dominant Languages are qualitatively similar. In the same vein, Bernardini and Schlyter (2004) noted that the developmental trajectory of the Weaker Language of simultaneous Swedish–Italian/German bilingual children followed the same milestones as in the Dominant Language, but the lexical realization was delayed. Several recent studies investigating language development in a simultaneous bilingual child with the Weaker HL-Russian and the Dominant SL-Turkish show that despite reduced input in HL-Russian, the acquisition of grammatical categories in the Weaker HL-Russian (e.g., aspect marking, case morphology and grammatical gender assignment) follows the same pattern as in monolingual acquisition (Antonova Ünlü and Li, 2016, 2017, 2018).

In contrast, there is also evidence that morpho-syntactic abilities of unbalanced bilinguals in their Weaker Language differ not only quantitatively, but also qualitatively as compared to monolingual children (Müller and Hulk, 2001; Paradis and Navarro, 2003; Argyri and Sorace, 2007; Kupisch, 2007; Sorace and Serratrice, 2009; Ringblom, 2012; Meir et al., 2017; Dobrova and Ringblom, 2018). This line of research supports the Deviance Hypothesis, suggesting that the Weaker Language of bilinguals is influenced by the Dominant Language. For example, Ringblom (2012), based on the longitudinal data of a simultaneous bilingual Russian–Swedish child, concluded that the development of the Weaker Language (HL-Russian) did not always follow a monolingual trajectory and was strongly influenced by the Dominant Swedish. The acquisition of rich morphology in the Weaker HL-Russian was reported to be challenging in contact with SL-Swedish which has sparse inflectional morphology. As for complex syntactic development of the Weaker HL-Russian, the errors produced by the child clearly suggest that the Weaker Language heavily relies on the Dominant language. The production of relative clauses in the Weaker HL-Russian was supported by the Dominant SL-Swedish: *eto ja som sdelal eto* ‘this I who did this,’ where the Russian *wh*-pronoun which should be inflected for case, gender and number is replaced by a Swedish uninflected complementizers *som* (see Dobrova and Ringblom, 2018).

Rodina and Westergaard (2017) showed that Russian–Norwegian bilinguals with two Russian-speaking parents show similar performance to monolinguals on gender agreement/assignment in HL-Russian. However, the bilinguals with the Weaker HL-Russian, who grew in one-parent-one-language families, showed not only a quantitative disadvantage as compared to monolinguals but also a different error profile. Bilinguals with the Weaker HL-Russian predominantly used masculine agreement across the board, this error pattern is neither observed in monolinguals nor in BB.

A recent study by Janssen (2016) investigated the acquisition of nominal morphology by Dutch-Dominant bilinguals with HL-Russian and HL-Polish as their Weaker Languages in comparison with BB and bilinguals in the Dominant Language. Dutch-Dominant bilinguals had more difficulties with case morphology and gender agreement/assignment in their Weaker HL-Polish and HL-Russian as compared to BB and bilinguals in the Dominant Language. Problems with case morphology in HL-Polish and HL-Russian can be attributed to the influence of the Dominant SL-Dutch which does not use nominal inflections. Yet, error profiles were not compared across the bilingual groups.

To sum up, most previous studies addressing the Weaker Language development have compared unbalanced bilinguals to monolinguals (but see Müller and Kupisch, 2003; Bernardini and Schlyter, 2004). Rather than comparing monolingual and bilingual grammars, the current study will probe whether grammatical representations in the Weaker Language are similar/different to those of BB and unbalanced bilinguals in the Dominant Language. Few studies investigated error profiles of unbalanced bilinguals in their Weaker Language as compared to BB and unbalanced bilinguals in the Dominant Language to determine whether the Weaker Language is delayed or deviant from other bilinguals. In this study, deviance is viewed as diverging from other bilingual patterns of acquisition, rather than from monolingual ones. Moreover, the assumption of the current study is that that the Weaker Language development in bilinguals with TLD, whether delayed or affected by the Dominant Language, is not disordered. Previous findings show that there are quantitative and qualitative differences between monolingual children with SLI and bilinguals with TLD (e.g., Paradis et al., 2008; Armon-Lotem, 2014). Thus, comparison of linguistic profiles of unbalanced bilinguals in the Weaker Language and bilinguals with SLI will shed light on the developmental trajectories of both populations.

### Morpho-Syntactic Abilities in Children With Specific Language Impairment (SLI): Delayed or Deviant

Children with SLI exhibit a primary deficit in language, in the absence of documented neurological damage, hearing deficits, severe environmental deprivation, or mental retardation (Tomblin et al., 1997; Leonard, 2014). Bilingual children with SLI show deficits in both of their languages (Håkansson et al., 2003; Armon-Lotem and de Jong, 2015; Thordardottir, 2015). Similarly to the Weaker Language development, language development in children with SLI has been discussed in terms of delay and/or deviance (for an overview see Leonard, 2014). Delay suggests a typical pattern of acquisition, while deviance stands for disordered/atypical trajectory of language development.

Most studies addressing the delay-deviance debate have compared monolingual children with SLI to younger language-matched children with TLD (matched by MLU, vocabulary, grammar, general language skills). The Delay Hypothesis is reinforced by the findings that children with SLI have a late start, their language development is protracted, and their error patterns are typical of younger children with TLD. For example, Rice et al. (1995), showed similarities in the acquisition of verbal morphology between monolingual children with SLI and younger children with TLD. For morpho-syntax, monolingual children with SLI were reported to perform similarly to younger language-matched controls (Stokes et al., 2006). The opposing view, the Deviance Hypothesis, has been advanced in studies reporting different error profiles in monolingual children with SLI and younger children with TLD. For instance, children with SLI have been shown to produce more bare stems compared to younger language-matched children in contexts, which require inflected forms (e.g., Bishop, 2014). Similarly, there are findings on morpho-syntactic abilities demonstrating distinct error profiles for children with SLI and younger language-matched controls (Briscoe et al., 2001; Riches, 2012). Moreover, it has been shown that language deficits in children with SLI may persist into adolescence (e.g., Conti-Ramsden et al., 2012), which would argue against the Delay Hypothesis or at least suggest that the initial delay becomes, in the long run, a deviance.

As for studies on bilingual children with SLI, the delay-deviance debate has not been addressed. Previous research shows similarities in linguistic profiles of monolingual and bilingual children with SLI, suggesting that disordered language development is similarly manifested irrespective of language status of a child (monolingual or bilingual). For example, Boerma et al. (2017) showed similarities in error profiles for participial affix use in Dutch among monolingual and bilingual children with SLI. Subject-verb agreement in German was reported to be similarly difficult for monolingual and bilingual children with SLI (Rothweiler et al., 2017). Russian–Hebrew speaking bilingual children with SLI were found to have difficulties with wh-questions and relative clauses (Meir et al., 2016) similarly to monolingual Hebrew speaking children with SLI (e.g., Friedmann and Novogrodsky, 2004; Novogrodsky and Friedmann, 2006). Similarly, monolingual and bilingual children with SLI showed difficulties with complex structures in German: wh-questions, relative clauses, embedding and finite complement clauses (Abed Ibrahim and Hamann, 2017; Hamann and Abed Ibrahim, 2017). In the same vein, monolingual and bilingual French speaking children with SLI were reported to have similar difficulties with morphology and syntax (Fleckstein et al., 2018).

To recap, the delay-deviance debate regarding language acquisition in children with SLI is still open. On the one hand, there are findings showing that children with SLI do not differ from younger language-matched controls which brings support to the claim that SLI is a delay. Conversely, there are studies showing quantitative and qualitative differences between children with SLI and younger children with TLD arguing for the Deviance Hypothesis.

### The Current Study

The present study has two aims. First, it attempts to advance our knowledge on the Weaker Language of unbalanced bilingual children with TLD. Furthermore, the study aims to bring new evidence for the delay-versus-deviance debate for the Weaker Language development in unbalanced bilingual children with TLD and for language acquisition in children with SLI. The following research questions are addressed in the study:

(1) To what extent there are quantitative and qualitative differences between unbalanced bilingual children with TLD in the Weaker Language and BB, on the one hand, and unbalanced bilinguals in the Dominant Language, on the other hand.
(2) To what extent there are quantitative and qualitative differences between bilinguals with atypical language development (i.e., SLI) and unbalanced bilinguals with TLD in the Weaker Language.

Error profiles across different bilingual groups are expected to shed light on the nature of the Weaker Language development. It is hypothesized that similarities in error profiles of unbalanced bilinguals in the Weaker Language and BB and bilinguals in the Dominant Language would point at commonalities of language development in bilinguals with TLD irrespective of their dominance status. Differences in error profiles are hypothesized to signify different developmental patterns in the Weaker Language as compared to BB and bilinguals in the Dominant Language.

As for language development in children with SLI, it is hypothesized that similar linguistic profiles of unbalanced bilinguals in the Weaker Language and bilinguals with SLI would point at typical patterns of bilingual acquisition in the two populations favoring the Delay Hypothesis. By contrast, differences between bilinguals with SLI and unbalanced bilinguals in the Weaker Language would point at a disorder, rather than a delay, in children with SLI.

## Materials and Methods

### Participants

For the purposes of the current study, one hundred and nineteen children aged 5;5–6;5 were drawn from a larger pool of participants (Meir, 2017). All bilingual children were recruited from regular and language preschools with SL-Hebrew as the language of instruction. All bilingual children were born in Israel to Russian-speaking families and were exposed to Russian from birth and had at least 12 months of exposure to SL-Hebrew.

Four groups of bilinguals were compared: three groups of bilinguals with TLD [unbalanced bilinguals with the Weaker HL-Russian and the Dominant SL-Hebrew (HL-weak: *n* = 39), unbalanced bilinguals with the Weaker SL-Hebrew and the Dominant HL-Russian (SL-weak: *n* = 19); balanced bilinguals (BB: *n* = 38) and a group of bilingual children SLI (biSLI: *n* = 23)]. All children were tested on non-verbal IQ using Raven’s colored progressive matrices non-verbal IQ test (Raven, 1998).

Language dominance in the current study was determined by language proficiency scores in both languages, following previous research (Paradis et al., 2003; Bedore et al., 2012; Iluz-Cohen and Armon-Lotem, 2013; Lust et al., 2016). In HL-Russian, language proficiency was measured using the *Russian Language Proficiency Test for Multilingual Children* (Gagarina et al., 2010). The Russian proficiency test is comprised of a battery of expressive (noun/verb naming, production of case, and verb inflections) and receptive (comprehension of grammatical constructions, receptive vocabulary) subtests. In SL-Hebrew, language proficiency was tested using the *Goralnik Screening Test for Hebrew* (Goralnik, 1995). The Hebrew proficiency measure includes subtests for expressive vocabulary, sentence repetition, sentence comprehension, expression, pronunciation, and story-telling. Since proficiency measures in Russian and Hebrew were not parallel, provisional bilingual cut-off points were used (Altman et al., 2016), rather than subtracting scores in HL and SL.

Children with TLD were identified if there were no prior parental concern about their language development and scored within the bilingual norm in at least one of their languages (HL or SL). Children with TLD were assigned to the group of BB if they scored above -1.25 *SD* in both of their languages. Unbalanced bilinguals with TLD were identified if they showed discrepancies in the proficiency scores. Children who scored below -1.25 *SD* in HL-Russian, but above the cut-off point of -1.25 *SD* in SL-Hebrew were labeled as unbalanced bilinguals with the Weaker Russian (HL-weak). Children who scored below -1.25 *SD* in SL-Hebrew, but above the cut-off point of -1.25 *SD* in HL-Russian were labeled as unbalanced bilinguals with the Weaker Hebrew (SL-weak).

Bilingual children with SLI (biSLI) were identified if they scored below -1.25 *SD* in both languages using bilingual norms and had parent/teacher reported history of SLI/concerns about their language milestones or an evaluation by a certified SLP.

**Table 1** presents background information which was collected using a short version of the BIPAQ parental questionnaire (Abutbul-Oz et al., 2012). A one-way ANOVA showed that the four groups were matched for age [*F*(3,115) = 0.80, *p* = 0.49], socio-economic status as measured by maternal education in years [*F*(3,111) = 0.89, *p* = 0.45] and non-verbal IQ [*F*(3,115) = 0.04, *p* = 0.99].

**Table 1:** Background information (means and standard deviations) on the participants per group.

	BB (*N* = 38)	HL-weak (*N* = 39)	SL-weak (*N* = 19)	biSLI (*N* = 23)
Age (in months)	71 (3)	71 (2)	72 (2)	72 (4)
Mother’s education (in years)	15 (3)	14 (3)	14 (3)	14 (3)
Age of SL onset (in months)	37 (16)	23 (24)	47 (15)	38 (15)
Length of exposure to SL	34 (16)	48 (23)	26 (15)	34 (16)
Non-verbal IQ	113 (12)	113 (12)	114 (18)	113 (11)


*BB, Balanced bilinguals; HL-weak, bilinguals with the Weaker Russian and the Dominant Hebrew; SL-weak, bilinguals with the Weaker Hebrew and Dominant Russian; biSLI, bilinguals with SLI; SL, Societal Language.*

By definition, there were group differences in language proficiency scores in HL-Russian [*F*(3,115) = 79.93, *p* < 0.001, η^2^ = 0.68] and in SL-Hebrew [*F*(3,115) = 75.87, *p* < 0.001, η^2^ = 0.66] (see **Table 2**). Balanced and unbalanced bilinguals in the Dominant Language outperformed bilinguals in the Weaker Language and the biSLI group [in HL-Russian: (BB = SL-weak) > (HL-weak = biSLI); in Hebrew: (BB = HL-weak) > (SL-weak = biSLI)].

**Table 2:** Language proficiency scores per group.

	BB (*N* = 38)	HL-weak (*N* = 39)	SL-weak (*N* = 19)	biSLI (*N* = 23)
Proficiency in HL-Russian (raw score)	87 (7)	57 (15)	87 (7)	51 (15)
Proficiency in HL-Russian (*Z*-score score)	0.16 (0.77)	-3.34 (1.69)	0.11 (0.75)	-4.01 (1.67)
Proficiency in SL-Hebrew (raw score)	146 (11)	148 (13)	110 (15)	100 (22)
Proficiency in SL-Hebrew (*Z*-score score)	0.16 (0.75)	0.30 (0.83)	-2.24 (1.00)	-2.88 (1.46)
Vocabulary scores in HL-Russian (subtest of the proficiency in HL-Russian)	36 (5)	18 (8)	37 (5)	17 (8)
Vocabulary scores in SL-Hebrew (subtest of the proficiency in SL-Hebrew	16 (5)	15 (5)	8 (3)	10 (4)
Parental rating of HL-Russian (0–4 scale)^∗^	3.62 (0.55)	2.69 (0.86)	3.67 (0.49)	2.43 (0.68)
Parental rating of SL-Hebrew (0–4 scale)^∗^	2.95 (0.88)	3.42 (0.55)	2.50 (0.79)	2.38 (0.80)


*^∗^Information was missing for 1 child in the SL-weak group; 2 children in the biSLI group; 3 children in the HL-weak group and 1 child in the BB group.*

*BB, balanced bilinguals; HL-weak, bilinguals with the Weaker Russian and the Dominant Hebrew; SL-weak, bilinguals with the Weaker Hebrew and Dominant Russian; biSLI, bilinguals with SLI; HL, Heritage Language; SL, Societal Language.*

Similarly to language proficiency scores, there were group differences in AoO [*F*(3,115) = 7.82, *p* < 0.001, η^2^ = 0.17]: [(BB = biSLI = SL-weak) < HL-weak]. Since length of exposure to SL-Hebrew is computed by deducting the AoO from the chronological age, similarly to AoO differences, there were significant differences for LoE [*F*(3,115) = 7.27, *p* < 0.001, η^2^ = 0.16]: [(BB = biSLI = SL-weak) > HL-weak]. Previous studies have used exposure measures as a proxy of language dominance. This study also shows that exposure measures (AoO and LoE) are linked to unbalanced bilingualism: unbalanced bilinguals with HL-weak and with SL-weak differed on AoO and LoE.

Since many studies, determine language dominance based on the discrepancy in the vocabulary size, expressive vocabulary scores for this sample are reported in **Table 2**. There was a group effect for HL-Russian [*F*(3,115) = 79.21, *p* < 0.001, η^2^ = 0.67] and for SL-Hebrew [*F*(3,115) = 36.65, *p* < 0.001, η^2^ = 0.49]. Follow-up pair-wise comparisons using Tamhane-2 *post hoc* tests for unequal variances revealed the following differences for HL-Russian: (BB = SL-weak) > (HL-weak = biSLI). Bonferroni *post hoc* tests showed a similar picture for SL-Hebrew: (BB = HL-weak) > (SL-weak = biSLI).

Parental ratings of the child’s language skills in HL-Russian and SL-Hebrew were also noted using a 4-point scale: 1(poor) – 4(very good) (see **Table 2**). Correlational analysis revealed significant correlations between parental ratings and proficiency scores for HL-Russian [*r*(112) = 0.72, *p* < 0.001] and SL-Hebrew [*r*(112) = 0.53, *p* < 0.001]. Similarly to the results for the proficiency scores, the analysis of parental ratings indicated that there were significant group differences in HL-Russian [*F*(3,108) = 22.72, *p* < 0.001, η^2^ = 0.39] and SL-Hebrew [*F*(3,108) = 10.54, *p* < 0.001, η^2^ = 0.32]. Parental ratings converged with the direct assessment measures for HL-Russian ((BB = SL-weak) > (HL-weak = biSLI)). In SL-Hebrew, the biSLI group and the SL-weak group obtained similar ratings (*p* = 1.00). The biSLI group received significantly lower scores than the BB and the HL-weak groups (*p* = 0.045, *p* < 0.001; respectively). Interestingly, SL-Hebrew parental ratings of the BB with TLD were similar to those of the SL-weak and the HL-weak (*p* = 0.26, *p* = 0.06; respectively). These findings indicate that parents of BB children under-estimate their children’s abilities in the SL. This can be explained by the fact that BB, who have good language skills in both of their languages, conduct their communication in the HL with the parents; and maybe the parents did not master the SL themselves and cannot evaluate their children’s ability in the SL.

### Procedure and Materials

The study was approved by Bar-Ilan University’s IRB and by the Israeli Ministry of Education. Prior to the study, parental written consent forms were secured. Before each session, child assent was obtained. Each participant was tested individually in a quiet room at preschools. Testing was performed by native speakers of each language.

Sentence Repetition (SRep) tasks in Russian and in Hebrew were administered in two separate sessions, on different days. The order of language sessions (HL-Russian first, SL-Hebrew first) was counter-balanced. The experimental tasks were pre-recorded by native speakers of Russian and Hebrew for the consistency of presentation and were presented via a power-point presentation using earphones. The participants were instructed to repeat the stimuli orally verbatim. Practice items preceded the experimental items to ensure that the child understood the task.

The SRep tasks in Russian (Meir and Armon-Lotem, 2015) and in Hebrew (Meir et al., 2016) were based on LITMUS-SRep (Marinis and Armon-Lotem, 2015) developed within COST Action IS0804^1^ and contained 56 sentences in each language (see **Tables A1**, **A2** in **Appendix A**). The Russian and the Hebrew tasks elicit SVO sentences, biclausal sentences with coordination and subordination, object and oblique questions, object relatives and conditionals (real and unreal). The Russian SRep task additionally includes simple SOV and OVS sentences and subject relatives. The Hebrew SRep task additionally includes simple VSO sentences, oblique relative clauses and biclausal sentences with phrasal conjunctions. Following Marinis and Armon-Lotem (2015), the children’s repetitions of the sentences were scored as correct if target structures were correctly reproduced. This scoring method enables to assess morpho-syntactic abilities of bilingual children without penalizing them for vocabulary errors. The proportion of correctly repeated structure out of 56 was calculated. Lexical substitutions were scored as correct (e.g., brother/boy, soup/food).

Furthermore, morphological accuracy was noted. Russian and Hebrew bilingualism offers an excellent opportunity to examine cross-linguistic influence, since the two languages vary in their selection of grammatical categories and vary in their mapping. For example, definiteness has an overt realization in Hebrew but not in Russian; aspect is realized in Russian but not in Hebrew. [ACC] case is realized in both languages, yet [ACC] case is differently mapped onto lexical categories in the two languages: in Russian [ACC] case is mapped onto nominal inflections, while in Hebrew [ACC] case is realized with the dedicated [ACC] marker *et* before [DEF] nouns. In Russian and in Hebrew verbal inflections mark categories of [Person], [Number], and [Gender].

A comparison of these morphological markings enables a fine-grained linguistic analysis in addressing directionality of cross-linguistic influence in bilingual children. The proportion of errors out of the total elicited items was calculated for each grammatical category. For example, in Russian and in Hebrew, verbal errors were analyzed in sentences in which verbs and overt subjects were produced. Sentences with null subjects were not included in the analysis. Erroneous use of [Person], [Number], [Gender] was noted: *ha-imahot ^∗^SHOTIM qafe* ‘the mothers.PL.FEM drink.PL.MASC coffee’; *mama ^∗^POZVONIL* ‘mother called.MASC’. Omissions of the definite marker *ha-* were noted only if the noun was produced: *imahot* ‘mothers’ instead of the targeted DP *ha-imahot* ‘DEF mothers.’ In Russian, erroneous use of the imperfective aspect marking was noted only on the elicited verbs: *tjotja ^∗^MYLA posudu* ‘aunt washed.IMPERF dishes’ instead of *tjotja po-myla posudu* ‘aunt washed.PERF dishes.’ The same coding method was applied for coding [ACC] case errors on Russian nouns.

Furthermore, detailed error patterns analysis for each structure separately was conducted to in order to shed light on grammatical representations in bilingual children (for more details on the analysis see Meir et al., 2016).

### Statistical Analysis

The data analysis was carried out using SPSS Statistics Version 18.0. First, group differences for global SRep scores and performance in each structure in Russian and Hebrew were analyzed with one-way ANOVAs with group (HL-weak, SL-weak, BB, biSLI) as an independent variable. Further pair-wise comparisons were conducted using Bonferroni *post hoc* tests for equal variance or Tamhane-2 *post hoc* tests for unequal variance with an adjusted alpha-level for multiple comparisons. The equality of variance was determined using the Levene’s test.

To assess group differences on morphological markings, Kruskal–Wallis tests were applied with Mann–Whitney *U* tests as follow-ups for pair-wise comparisons. Finally, error profiles of unbalanced bilinguals in the Weaker Language were compared to those of bilingual children with SLI using Mann–Whitney tests.

## Results

### Findings for HL-Russian

#### Quantitative Comparison of the Four Bilingual Groups in HL-Russian

**FIGURE 1:**
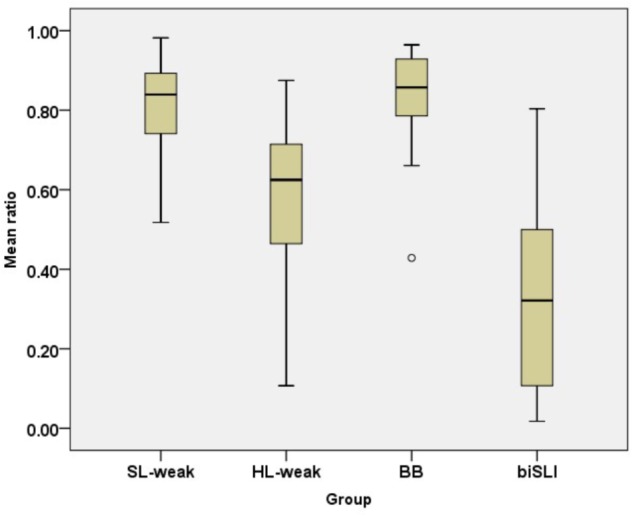
Box plots for scores on the on the SRep task in HL-Russian per group. The plots show the median (thick line within box), 25th and 75th percentiles (box), 10th and 90th percentiles (whiskers). Asterisks (^∗^) and Circles (∘) mark outliers and extreme cases.

**Figure 1** presents the performance on the SRep task in HL-Russian for the four bilingual groups. The analysis using a one-way ANOVA with children’s scores on the SRep task in Russian as a dependent variable and group (HL-weak, SL-weak, BB, biSLI) as an independent variable showed a significant effect of group [*F*(3,112) = 51.83, *p* < 0.001, η^2^ = 0.68]. Pair-wise comparisons using Tamhane-2 *post hoc* tests showed that the HL-weak group scored lower than BB and unbalanced bilinguals in the Dominant Language, yet higher the biSLI group: (BB = SL-weak) > HL-weak > biSLI (all *p-*values at *p* < 0.001).

Subsequently, the four groups were compared on 11 structures: group differences were detected for each structure (see **Table 3**). As determined by Tamhane-2 *post hoc* tests, the BB and the SL-weak groups scored similarly on all the structures. As for the HL-weak group, the comparison of their scores to the BB group showed a disadvantage on 9 out 11 structures; no differences were found on biclausal sentences with coordination and subordination. Similarly, the HL-weak group scored lower than the SL-weak group on 8 out of 11 structures; no differences were found only for biclausal sentences with coordination and subordination and OVS sentences. As for the comparison of the HL-weak and the biSLI the analysis showed that the HL-weak group outperformed the biSLI group on 7 out of 11 structures. There were no significant differences between the two groups on four syntactic structures: biclausal sentences with coordination, object questions, object relatives and real conditionals.

**Table 3:** Mean (*SD*) proportion of accuracy on 11 target structures in HL-Russian per group.

	BB (*N* = 38)	HL-weak (*N* = 37)	SL-weak (*N* = 38)	biSLI (*N* = 22)	*F*-results	η^2^*-value*
SVO	0.98 (0.08)	0.84 (0.18)	0.99 (0.04)	0.64 (0.29)	21.31^∗∗^	0.36
SOV	0.72 (0.27)	0.43 (0.26)	0.58 (0.21)	0.19 (0.20)	23.79^∗∗^	0.39
OVS	0.92 (0.17)	0.77 (0.26)	0.93 (0.11)	0.40 (0.33)	27.54^∗∗^	0.42
Biclausal sentences with coordination	0.99 (0.06)	0.84 (0.24)	0.95 (0.10)	0.59 (0.40)	14.95^∗∗^	0.29
Biclausal sentences with subordination	0.97 (0.08)	0.83 (0.31)	0.97 (0.08)	0.40 (0.38)	28.95^∗∗^	0.44
Object questions	0.88 (0.18)	0.55 (0.29)	0.80 (0.21)	0.38 (0.32)	23.24^∗∗^	0.38
Oblique questions	0.95 (0.12)	0.63 (0.33)	0.97 (0.08)	0.22 (0.29)	54.27^∗∗^	0.59
Subject relatives	0.80 (0.18)	0.51 (0.28)	0.75 (0.22)	0.24 (0.31)	28.43^∗∗^	0.43
Object relatives	0.57 (0.28)	0.23 (0.26)	0.57 (0.32)	0.13 (0.21)	20.07^∗∗^	0.35
Real conditionals	0.96 (0.12)	0.70 (0.35)	0.93 (0.14)	0.55 (0.43)	12.66^∗∗^	0.25
Unreal conditionals	0.61 (0.36)	0.16 (0.26)	0.63 (0.38)	0.03 (0.09)	28.87^∗∗^	0.44


*^∗∗^Significance at p < 0.001. BB, balanced bilinguals; HL-weak, bilinguals with the Weaker HL-Russian and the Dominant SL-Hebrew; SL-weak, bilinguals with the Weaker SL-Hebrew and Dominant HL-Russian; biSLI, bilinguals with SLI; SVO, Subject–Verb–Object; SOV, Subject–Object–Verb; OVS, Object–Verb–Subject.*

**FIGURE 2:**
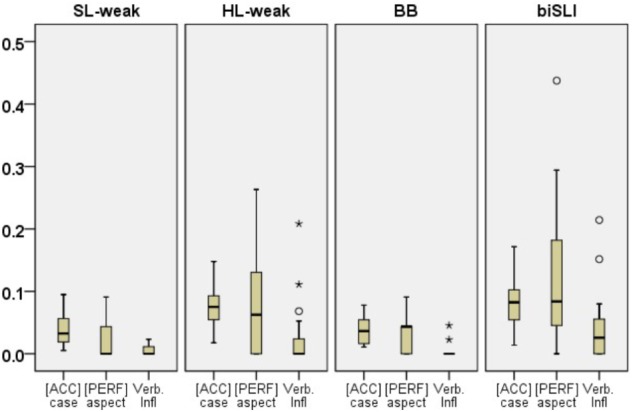
Box plots for proportions of errors for [ACC] case, [PERF] aspect and verbal inflections in HL-Russian per group. The plots show the median (thick line within box), 25th and 75th percentiles (box), 10th and 90th percentiles (whiskers). Asterisks (^∗^) and Circles (∘) mark outliers and extreme cases.

Further analysis compared morphological accuracy in HL-Russian across the four groups (see **Figure 2**). The Kruskal–Wallis test showed a group effect for [ACC] case errors [χ^2^(3) = 39.88, *p* < 0.001], [PERF] aspect [χ^2^(3) = 21.53, *p* < 0.001] and verbal inflections [χ^2^(3) = 31.41, *p* < 0.001]. Further pair-wise comparisons using Mann–Whitney *U* tests showed no differences for the BB and the SL-weak on [ACC] case (*U* = 56, *p* = 0.93), and [PERF] aspect (*U* = 331, *p* = 0.58), yet there were differences between the two groups on verbal inflections (*U* = 287, *p* = 0.03) with the BB group being more accurate on verbal inflections. The HL-weak group was less accurate on [ACC] case and [PERF] aspect than the BB (*U* = 01, *p* < 0.001; *U* = 427, *p* < 0.001, respectively) and the SL-weak groups (*U* = 137, *p* < 0.001; *U* = 200, *p* = 0.01, respectively). Yet, on verbal inflections the HL-weak showed marginal differences with the SL-weak group (*U* = 265, *p* = 0.08) and significant differences from the BB group (*U* = 432, *p* < 0.001). The comparison of the HL-weak and the biSLI groups showed no significant differences between the two groups for [ACC] case errors (*U* = 317, *p* = 0.16) and for [PERF] aspect errors (*U* = 354, *p* = 0.27). Group differences were observed for verbal inflection errors (*U* = 291, *p* = 0.03) with the biSLI group being less accurate.

#### Comparison of Morpho-Syntactic Profiles in HL-Russian

Subsequently, error profiles of the four bilingual groups were investigated. No differences were detected between the BB group and the SL-weak. Despite quantitative differences between the BB and the HL-weak groups, error profiles of the two groups seem to overlap. The only pattern which differentiated the HL-weak group from the BB group was the substitution of the *wh*-pronoun (inflected for case, number, and gender) with the non-declinable complementizer ‘*čto*’ in subject and object relative clauses (both comparisons at *p* < 0.001). This error might be attributed to the influence of Dominant-Hebrew, which uses non-declinable complementizer ‘*še*’ in subject and object relatives.

As for the comparison of the unbalanced bilinguals in their Weaker Language (HL-weak) and the biSLI, different error profiles emerged across several structures (see **Table 4**). The biSLI group produced more sentence fragments, omitted conjunctions and simplified structures (e.g., produced simple SVO sentences instead of targeted object questions, object relatives and subject relatives). Interestingly, the HL-weak group and the biSLI had similar accuracy scores on object relatives, yet error analysis showed that the underlying difficulties were of different natures [see Example (1)].

**Table 4:** Proportions of most prominent syntactic error patterns observed on SRep in HL-Russian (HL-weak vs. biSLI).

Target structure	Error type	HL-weak Mean	biSLI Mean	*U*-values (Mann–Whitney test)	*p*-value (Mann–Whitney test)
Biclausal with coordination	Sentence fragment	0.03	0.13	343	*p* = 0.006
Biclausal with subordination	Conjunction omission	0.04	0.27	242	*p* < 0.001
Real conditional	Conjunction omission	0.01	0.14	334	*p* < 0.001
Unreal conditional	Conjunction omission	0.01	0.08	352	*p* = 0.035
Oblique question	Preposition omission	0.08	0.31	242	*p* = 0.001
Object question (OQ)	OQ into SVO	0.04	0.16	291	*p* = 0.007
Object relative (OR)	OR into SVO	0.04	0.33	161	*p* < 0.001
	OR into wh-question	0.00	0.02	351	*p* = 0.006
	Case error	0.34	0.12	224	*p* = 0.001
Subject relative (SR)	SR into SVO	0.06	0.40	201	*p* < 0.001
	SR into wh-question	0.00	0.04	342	*p* = 0.011


*HL-weak, bilinguals with the Weaker HL-Russian and the Dominant SL-Hebrew; biSLI, bilinguals with SLI; SR, subject relatives; OR, object relatives.*

Children in the HL-weak group attempted to re-produce a complex structure [see Examples (1)]. Some HL-weak had difficulties with case inflections, producing both elements either in [NOM] or [ACC] (see 1a and 1b), some children substituted an inflected *wh-*pronoun with a non-declinable complementizer (the Hebrew ***še*** or the Russian ***čto***) (see 1c and 1d). Conversely, children in the biSLI group turned object relatives into simple SV or SVO sentences (see 1g–h).

**Table:** 

(1)	Target	*jeto*	*devočka*	*kotor-uju*	*narisovala*	*mama*		
		this	girl._NOM_	who-F.SG.ACC	drew.PERF	mother.NOM			
		‘This is the girl that the mother drew.’				
Responses of the children in the Weak-RUS group						
	(a)	*jeto*	*devočka*	*kotor-****aja***	*risovala*	*mam-a*		
		this	girl._NOM_	who-**_F.SG.NOM_**	drew._IMPERF_	mother._NOM_		
	(b)	*jeto*	*devočka*	*kotor-****uju***	*risovala*	*mam-a*		
		this	girl._NOM_	who-**_F.SG.ACC_**	drew._IMPERF_	mother._NOM_		
	(c)	*jeto*	*devočka*	***še***	*narisovala*	*mam-a*		
		this	girl._NOM_	that (Hebrew complementizer)	drew._PERF_	mother._NOM_		
	(d)	*jeto*	*devočka*	***čto***	*narisovala*	*mam-****u***		
		this	girl._NOM_	that**(**complementizer**)**	drew._PERF_	mother._ACC_		
	(e)	*jeto*	*devočka*	***kak-oj***	*narisovala*	*mam-****u***		
		this	girl._NOM_	which-***_M.SG.NOM_*** **(**wh-word**)**	drew._PERF_	mother._ACC_		
Responses of the children in the biSLI group						
	(f)	*jeto*	*devočka*	*narisovala*				
		this	girl._NOM_	drew._PERF_				
	(g)		*devočka*	*narisovala*	*mam-****u***			
			girl._NOM_	drew._PERF_	mother.ACC			
	(h)	*jeto*	*devočka*	*narisovala*	*mam-****a***			
		this	girl._NOM_	drew._PERF_	mother.NOM			


To sum up, the results for HL-Russian have demonstrated quantitative differences between the Weaker Language of bilinguals (HL-weak) and BB and unbalanced bilinguals in the Dominant Language. The morphological accuracy of unbalanced bilinguals in the Weaker Language was lower than in BB and bilinguals in the Dominant Language. In the Weaker Language, the unbalanced bilinguals showed similar performance to the biSLI group for [ACC] case and [PERF] aspect (on the features that are differently configured in Russian and Hebrew). On verbal inflections, the HL-weak group scored lower than the BB and the SL-weak, yet HL-weak outperformed the biSLI. Even though the HL-weak and the BB/SL-weak groups showed quantitative differences, their error profiles overlapped for most structures. The only error pattern which differentiated the two groups was wh-pronoun substitution with the complementizers in Russian or in Hebrew (e.g., ‘*čto*’/‘*še*’): this error pattern can be traced back to the influence of the Dominant-Hebrew.

Despite similar vocabulary scores in HL- Russian, the HL-weak group outperformed the biSLI group on the global SRep score and on a variety of structures. Both groups (HL-weak and biSLI) showed low accuracy on morphological categories that are differently configured in Russian and Hebrew (e.g., [ACC] case and [PERF] aspect). Importantly, error profiles of the HL-weak and the biSLI group were found to bear fundamental differences. While children in the biSLI simplified complex structures, unbalanced bilinguals in the Weaker Language opted for complex structures.

### Findings for SL-Hebrew

#### Quantitative Comparison of the Four Bilingual Groups in SL-Hebrew

**FIGURE 3:**
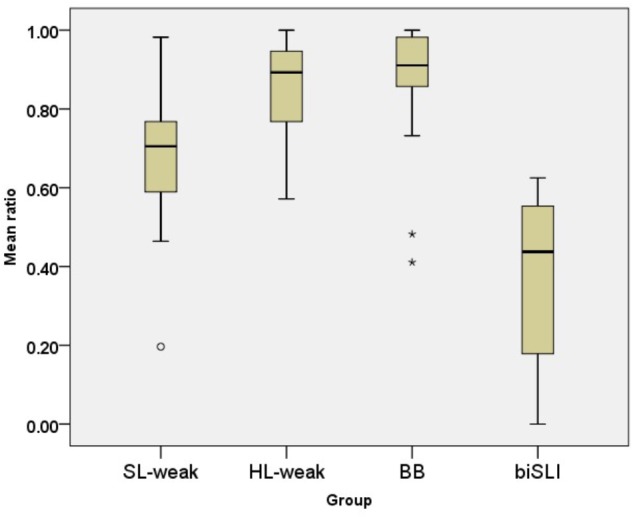
Box plots for the SRep task in SL-Hebrew per group. The plots show the median (thick line within box), 25th and 75th percentiles (box), 10th and 90th percentiles (whiskers). Asterisks (^∗^) and Circles (∘) mark outliers and extreme cases.

Turning to the SL-Hebrew data, **Figure 3** presents the performance on the SRep task in SL-Hebrew. The analysis using a one-way ANOVA with children’s scores on the SRep task in Hebrew as a dependent variable and group (HL-weak, SL-weak, BB, biSLI) as an independent variable showed a significant effect of group [*F*(3,112) = 64.69, *p* < 0.001, η^2^ = 0.63]. Follow-up pair-wise comparisons using Tamhane-2 *post hoc* showed that, similarly to the Russian data, unbalanced bilinguals with TLD in their Weaker Language scored lower than BB and bilinguals in the Dominant Language, yet higher than bilinguals with SLI [(BB = HL-weak) > SL-weak > biSLI].

Further analyses compared the performance of the four groups across the 11 structures of the Hebrew SRep Task (see **Table 5**). There was a group effect for all the structures. Follow-up pair-wise comparisons using Tamhane-2 *post hoc* tests showed that the BB and the HL-weak groups scored similarly across all the structures. The SL-weak scored lower than the BB group on 6 of 11 structures: no differences were observed for 5 structures (SVO, biclausal with coordination, biclausal with subordination, oblique questions, and object relatives). Similarly, the SL-weak scored lower than the HL-weak, i.e., bilinguals with the Dominant SL-Hebrew and the Weaker HL-Russian, on 7 out of 11 structures: no differences were detected for 4 structures (SVO, biclausal with coordination, biclausal with subordination, and object relatives). The comparison of the SL-weak and the biSLI group showed that the unbalanced bilinguals in the Weaker SL-Hebrew outperformed the biSLI group on 9 out of the 11 tested structures, no differences between the two groups were found for the unreal conditionals and biclausal sentences with coordination.

**Table 5:** Mean (*SD*) proportion of accuracy on 11 target structures in SL- Hebrew per group.

	BB (*N* = 38)	HL-weak (*N* = 39)	SL-weak (*N* = 18)	biSLI (*N* = 22)	*F*-results	η^2^-value
SVO	0.98 (0.04)	0.96 (0.07)	0.94 (0.13)	0.70 (0.26)	25.59^∗∗^	0.41
Biclausal sentences with coordination)	0.90 (0.15)	0.93 (0.17)	0.74 (0.33)	0.48 (0.41)	17.73^∗∗^	0.32
Biclausal sentences with subordination	0.97 (0.10)	0.96 (0.12)	0.83 (0.26)	0.47 (0.40)	29.15^∗∗^	0.44
Object questions	0.83 (0.25)	0.79 (0.25)	0.54 (0.29)	0.27 (0.27)	26.58^∗∗^	0.41
Oblique questions	0.91 (0.18)	0.83 (0.25)	0.67 (0.33)	0.14 (0.24)	54.41^∗∗^	0.59
Object relatives	0.93 (0.16)	0.93 (0.15)	0.85 (0.24)	0.45 (0.39)	23.88^∗∗^	0.39
Oblique relatives	0.94 (0.15)	0.96 (0.11)	0.72 (0.30)	0.35 (0.37)	42.56^∗∗^	0.53
VSO	0.88 (0.18)	0.83 (0.20)	0.69 (0.21)	0.41 (0.30)	24.27^∗∗^	0.39
Real conditionals	0.95 (0.10)	0.91 (0.15)	0.74 (0.25)	0.45 (0.33)	33.54^∗∗^	0.47
Unreal conditionals	0.76 (0.24)	0.68 (0.31)	0.35 (0.34)	0.16 (0.27)	25.57^∗∗^	0.40
Biclausal sentences with phrasal conjunctions	0.72 (0.28)	0.73 (0.27)	0.37 (0.31)	0.09 (0.12)	37.10^∗∗^	0.50


*^∗∗^Significance at p < 0.001.*

*BB, Balanced bilinguals; HL-weak, bilinguals with the Weaker HL-Russian and the Dominant SL-Hebrew; SL-weak, bilinguals with the Weaker SL-Hebrew and Dominant HL-Russian; biSLI, bilinguals with SLI; SVO, Subject–Verb–Object; VSO, Verb–Subject–Object.*

**FIGURE 4:**
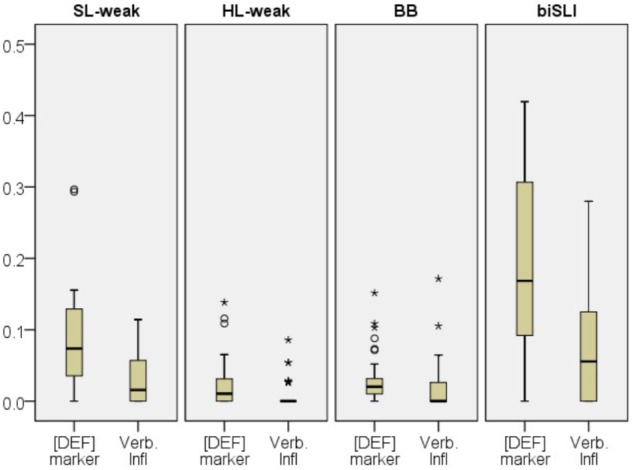
Box plots for proportions of errors for [DEF] marker and verbal inflections in SL-Hebrew per group. The plots show the median (thick line within box), 25th and 75th percentiles (box), 10th and 90th percentiles (whiskers). Asterisks (^∗^) and Circles (∘) mark outliers and extreme cases.

Subsequently, morphological accuracy (proportion of errors for the [DEF] marker *ha-* and verbal inflections) was compared across the four groups (see **Figure 4**). The Kruskal–Wallis test showed a group effect for [DEF] marking errors [χ^2^(3) = 42.928, *p* < 0.001] and verbal inflections [χ^2^(3) = 23.16, *p* < 0.001].

A Mann–Whitney *U* test indicated no significant differences between the BB and the HL-weak group for both morphemes ([DEF] marker: *U* = 647, *p* = 0.33, verbal inflections: *U* = 660, *p* = 0.29). The SL-weak group showed lower accuracy than the BB (*U* = 158, *p* < 0.001) and the HL-weak on [DEF] marker (*U* = 140, *p* < 0.001). On verbal inflections the SL-weak also showed lower performance than the HL-weak (*U* = 228, *p* = 0.01) and marginally lower than the BB group (*U* = 256, *p* = 0.08). As for the SL-weak and the biSLI group comparisons, the analysis showed significantly more omission of the [DEF] marker *ha-* in the biSLI group (*U* = 100, *p* = 0.01), and no differences between the two groups for the verbal inflections (*U* = 134, *p* = 0.12).

#### Comparison of Error Profiles in SL-Hebrew

Error pattern analysis showed that the BB group and the HL-weak group, i.e., unbalanced bilinguals who are dominant in SL-Hebrew, showed identical error profiles in their SL-Hebrew. Moreover, no differences were detected for error profiles of the SL-weak group and BB. However, differences in error profiles emerged between the SL-weak and the biSLI groups. **Table 6** presents the most prominent error patterns for the two groups (biSLI vs. SL-weak). Similarly to the Russian data, in SL-Hebrew the biSLI group turned complex sentences into simpler sentences (e.g., object questions were turned into simple SVO sentences) and had significantly more preposition and conjunction omissions.

**Table 6:** Proportions of most prominent syntactic error patterns observed on SRep in SL-Hebrew (SL-weak vs. biSLI).

Target structure	Error type	SL-weak Mean	biSLI Mean	*U*-values (Mann–Whitney test)	*p*-value (Mann–Whitney test)
Oblique question (OQ)	OQ into subject question	0.01	0.18	82	*p* = 0.002
	Preposition omission	0.17	0.38	112	*p* = 0.030
Object relatives (OR)	OR into SVO	0.07	0.23	115	*p* = 0.037
Advanced conjunctions	Conjunction omission	0.12	0.30	105	*p* = 0.017


*SL-weak, bilinguals with the Weaker Hebrew and Dominant Russian; biSLI, bilingual children with SLI; OQ, Object Question; OR, Object Relative.*

As demonstrated in (2), the SL-weak group reproduced object relatives (see 2a–b) while children in the biSLI group simplified relative clauses and produced simple SVO sentences or subject relatives (see examples 2d–e).

**Table:** 

(2)	Target	*zot*	*ha- yalda*	*še*	*ha- iša*		*niška*	
		this	_DEF_- girl	that	_DEF_- woman		kissed	
		‘This is the girl that.’					
Responses of the children in the Weak-RUS group								
	(a)	*zot*	*__ yalda*	*še*		*__ iša*	*niška*	
		this	___ girl	that		__ woman	kissed	
	(b)	*zot*	*ha- yalda*	*še*		*__ yalda*	*niška*	
		this	_DEF_- girl	that		___ girl	kissed	
Responses of the children in the biSLI group								
	(a)	*zot*	*ha- yalda*	*niška*		*__ iša*		
		this	_DEF_- girl	kissed		__ woman		
	(b)	*zot*	*ha- yalda*	*še*		*niška*	*__ iša*	
		this	_DEF_- girl	that		kissed	*__* woman	


The findings for SL-Hebrew converge with the results for HL-Russian: unbalanced bilinguals with TLD in the Weaker Language (i.e., SL-weak) differ from BB and bilinguals in the Dominant Language only quantitatively, while error profiles of all bilinguals with TLD bear a striking resemblance. Yet, unbalanced bilinguals in the Weaker Language show quantitative and qualitative differences from the biSLI group. Unbalanced bilinguals outperform the biSLI group on a number of measures and show different profiles from the biSLI group. While the former succeeded in reproducing complex structures despite their limited vocabulary, the latter simplified complex structures.

## Discussion

The current study was devised to determine to what extent morpho-syntactic abilities of bilingual children with TLD in the Weaker Language differ from those of BB and bilinguals in the Dominant Language, on the one hand, and bilingual children with SLI, on the other hand. This study attempted to add to the on-going debate on the nature of grammatical representations and developmental trajectories among unbalanced bilinguals in the Weaker Language and bilingual children with SLI. To achieve this goal, different bilingual patterns of acquisition were investigated. This study addressed the delay-deviance hypothesis in bilingual children rather than comparing monolingual and bilingual trajectories of acquisition.

### The Weaker Language and the Balanced/Dominant Language of Bilinguals

The first research question of the current study aimed to explore morpho-syntactic manifestations in the Weaker Language of unbalanced bilinguals with TLD as compared to BB and bilinguals in the Dominant Language. Numerous studies have demonstrated quantitative differences between the Weaker Language of unbalanced bilinguals and BB and bilinguals in the Dominant Language (e.g., Schlyter, 1994; Jisa, 2000; Bernardini and Schlyter, 2004; Gutiérrez-Clellen et al., 2006; Bedore et al., 2011; Hoff et al., 2012). Yet, previous studies brought conflicting evidence with respect to qualitative differences between bilinguals in the Weaker Language and monolinguals and BB and bilinguals in the Dominant Language. Some studies have shown that the acquisition patterns in the Weaker Language are similar to the ones of BB and bilinguals in the Dominant Language and even monolinguals (e.g., Müller and Kupisch, 2003; Bernardini and Schlyter, 2004; Antonova Ünlü and Li, 2016, 2017, 2018). Alternatively, the Deviance Hypothesis was supported by findings indicating that grammars of unbalanced bilinguals in their Weaker Language differ qualitatively from the monolingual baseline grammars (e.g., Yip and Matthews, 2000; Argyri and Sorace, 2007; Ringblom, 2012; Janssen, 2016; Meir et al., 2017). Previous studies have brought convincing evidence that the two linguistic systems of a bilingual person are susceptible to bi-directional cross-linguistic influence: influence from HL onto SL and from SL onto HL (e.g., Ge et al., 2017; Hervé and Serratrice, 2017; Meir et al., 2017). This study has focused on different bilingual outcomes rather than comparing bilingual performance to a monolingual “golden standard.”

The results of the current study reiterate previous findings showing that the Weaker Language of unbalanced bilinguals is quantitatively poorer. Unbalanced bilinguals in the Weaker Language have smaller vocabularies and are less accurate on a variety of morpho-syntactic structures as compared to BB and bilinguals in the Dominant Language. Moreover, unbalanced bilinguals in the Weaker Language have more pronounced difficulties with morphology as compared to BB and bilinguals in the Dominant Language. For example, in HL-Russian unbalanced bilinguals with the Weaker Russian showed lower accuracy for [ACC] case marking and for [PERF] aspect marking in comparison with BB and bilinguals in the Dominant Language.

However, despite quantitative differences, error profiles of unbalanced bilinguals in the Weaker Language bore resemblance to the ones of BB and bilinguals in the Dominant Language, extending the finding by Müller and Kupisch (2003) and Bernardini and Schlyter (2004) to two different patterns of unbalanced bilingual acquisition: for the Weaker HL and the initial Weaker SL. Despite lower scores in HL-Russian on various syntactic structures, error patterns in unbalanced bilinguals with the Weaker HL-Russian were similar to those of BB and bilinguals who are Dominant in HL-Russian. That is all bilinguals with TLD had difficulties with case inflections. Previously, case inflectional morphology has been reported to pose difficulties to bilingual children who acquire Russian as their HL and SL that does not mark cases with inflections (Turian and Altenberg, 1991; Peeters-Podgaevskaja, 2008; Gagarina, 2011; Schwartz and Minkov, 2014; Janssen et al., 2015; Meir and Armon-Lotem, 2015). In HL-Russian, unbalanced bilinguals with the Weaker HL-Russian and the Dominant SL-Hebrew substituted the declinable *wh-*pronoun (marked for case, gender, and number) with the non-declinable complementizer ‘*čto*’ in subject and object relative clauses. This substitution can be easily attributed to the influence of the Dominant SL-Hebrew, since Hebrew utilizes non-declinable caseless complementizers ‘*še*’ in relative clauses. Moreover, such an error pattern has been previously reported for a child with the Weaker Russian and the Dominant Swedish: the child used a Swedish uninflected complementizers *som* in relative clauses instead of a Russian declinable *wh*-pronoun [e.g., *eto ja som sdelal eto* ‘this I who did this’ (see Dobrova and Ringblom, 2018)].

Similarly, in SL-Hebrew, there were quantitative differences between unbalanced bilinguals with the Weaker SL-Hebrew and BB and bilinguals with the Dominant SL-Hebrew. Bilinguals with the Weaker SL-Hebrew showed lower performance for the global score on the Hebrew SRep task. They also showed lower levels of accuracy on nearly half of the morpho-syntactic structures as compared to BB and bilinguals with the Dominant SL-Hebrew. Bilinguals with the Weaker SL-Hebrew were less accurate on morphological markings of definiteness and verbal inflections as compared to BB and bilinguals with the Dominant SL-Hebrew.

Yet, importantly, the analysis of error profiles of unbalanced bilinguals with the Weaker SL-Hebrew and BB and bilinguals in the Dominant Language showed that there are no differences between the groups. The results indicate that bilinguals with TLD who have mastered complex constructions in their HL seem to draw on their existing linguistic knowledge to produce complex structures in the SL, albeit their poor lexicons and morphology in the Weaker Language.

Future research exploring grammatical representations of unbalanced bilinguals with TLD should explore other language pairs in order to deepen our understanding on how typological differences affect grammatical representations in the Weaker Language under the influence of the Dominant Language. Research on unbalanced bilingualism should be extended to school-age children. This line of research would enable us to evaluate how grammatical representations of BB and unbalanced bilinguals with the Weaker HL, who get extensive exposure and acquire literacy skills in their SL, change over time.

### Delay-Deviance Debate: Unbalanced TLD in the Weaker Language and Atypical Language Development

The second research question of the study aimed to contribute to the delay-versus-deviance debate on language acquisition patterns of unbalanced bilinguals with TLD in the Weaker Language and bilinguals with SLI. Language development in unbalanced bilinguals in the Weaker Language is not expected to be disordered, while it may be delayed and or/influenced by the Dominant Language. Thus, it was hypothesized that similarities in the profiles of the Weaker Languages of bilinguals with TLD and bilinguals with SLI would point at similar morpho-syntactic representations in the two populations. Qualitative differences in error profiles of unbalanced bilinguals with TLD in the Weaker Language and bilinguals with SLI were predicted to support a deviant language acquisition pattern in children with SLI.

Despite similar vocabulary sizes of unbalanced bilinguals in the Weaker Language and bilinguals with SLI, the former showed higher scores on SRep tasks. More importantly, error profiles of the two populations were found to be different. Whereas unbalanced bilinguals in the Weaker Language opted for complex structures relying on the available resources from the Dominant Language, bilinguals with SLI opted for simplified structures. Bilinguals with SLI produced simple SVO sentences instead of targeted object questions, object relatives and subject relatives. This has been also found for monolingual children with SLI (e.g., Novogrodsky and Friedmann, 2006). Previous research has pointed at similarities in linguistic profiles of monolingual and bilingual children with SLI, suggesting that disordered language development is similarly manifested irrespective of language status of a child (monolingual or bilingual) (see Meir et al., 2016; Abed Ibrahim and Hamann, 2017; Boerma et al., 2017; Hamann and Abed Ibrahim, 2017; Rothweiler et al., 2017). Importantly, this has been confirmed for both languages of bilinguals with SLI (HL-Russian and SL-Hebrew).

The current study convincingly shows that grammatical representations in unbalanced bilinguals in the Weaker Language and bilinguals with SLI differ. The results for bilinguals with SLI couple with the literature on monolingual children with SLI suggesting that language profiles of children with SLI show a deviant pattern of acquisition (e.g., Briscoe et al., 2001; Conti-Ramsden et al., 2012; Riches, 2012; Bishop, 2014).

## Conclusion

The current study assessed grammatical representations of unbalanced bilinguals in the Weaker Language in the group of Russian–Hebrew speaking pre-school children. The findings indicate that grammatical representations of unbalanced bilinguals (either HL or SL) are qualitatively similar to the ones of BB and bilinguals in the Dominant Language, albeit their performance is quantitatively disadvantaged. The Weaker Language of bilinguals is characterized by an increased number of morphological errors, especially when the two languages (HL and SL) show differences in the selection and mapping of morpho-syntactic categories. The findings indicate that mastering rich morphology in the context of reduced input is challenging. Yet, despite limited vocabulary size and limited arsenal of morphological markings, unbalanced bilinguals attempt to derive complex structures in the Weaker Language recruiting resources from their Dominant Language.

The comparison of morpho-syntactic abilities of unbalanced bilinguals in the Weaker Language to those of bilinguals with SLI has demonstrated that the disordered pattern of acquisition is different from that of the Weaker Language development in unbalanced bilinguals with TLD. Whereas unbalanced bilinguals in the Weaker Language attempt to produce complex structures, relying on the available resources from the Dominant Language; bilinguals with SLI simplified structures.

## Ethics Statement

This study was approved by Bar Ilan University review board as well as by the Israeli Ministry of Education. Parents provided informed written consent in accordance with the Declaration of Helsinki.

## Author Contributions

NM developed the research questions, carried out the data analyses, and wrote the manuscript.

## Conflict of Interest Statement

The author declares that the research was conducted in the absence of any commercial or financial relationships that could be construed as a potential conflict of interest. The reviewer LI and handling Editor declared their shared affiliation.

## Footnotes

1 www.bi-sli.org

## APPENDIX A: Structures Tested in the Srep Tasks Based on Litmus-Srep Developed Within the Cost Action Is0804 (Marinis and Armon-Lotem, 2015).

**Table A1:** Structures tested in the Russian SRep task with examples (number of sentences per structure in brackets).

Level	Structure	Example						
1	SVO (8)	*mal’ciki*	*myli*	*posudu*	*posle obeda*.			
		boys.NOM	washed.PL	dishes.ACC	after lunch			
		‘A/The boys did dishes after lunch’						
	SOV (4)	*mal’cik*	*devocku*	*požalel*	*na uroke*			
		boy.NOM	girl.ACC	pitied.MASC.SG	on lesson
		‘A/The boy pitied a/the girl during the lesson’.						
	OVS (4)	*kota*	*uvidela*	*myška*	*vo dvore*.			
		cat.ACC	saw.FEM.SG	mouse.NOM	in yard			
		‘A/The mouse saw a/the cat in the yard’.						
2	Biclausal sentences	*mama*	*ispekla*	*tort*,	*i*	*my*	*pozvali*	*druzej.*
	with coordination (4)	mother.NOM	baked. FEM.SG	cake.ACC	and	we.NOM	called.PL	friends.ACC
		‘The mother made a cake and we invited friends’.						
	Biclausal sentences	*devočka*	*vzjala*	*zontik*,	*potomu čto*	*šjol*	*dožd’.*	
	with subordination (4)	girl.NOM	took.FEM.SG	umbrella	because	went.MASC.SG	rain.NOM	
		‘A/They girl took an umbrella because it was raining’						
	Object questions (4)	*kakuju*	*obez’janu*	*našjol*	*krokodil?*			
		which.FEM.ACC	monkey.FEM.ACC	found.MASC.SG	crocodile.MASC.NOM?			
		‘Which monkey did a/the crocodile find?’						
	Oblique questions (4)	*u*	*kakogo*	*malćika*	*sestra*	*vzjala*	*kraski?*	
		at	which.MASC.GEN	boy.GEN	sister.FEM.NOM	took.FEM.SG	paints.ACC	
		‘From which boy did the sister take paints?’						
3	Real conditionals (4)	*my poedem*	*na*	*more*,	*esli*	*prosnemsja*	*rano.*	
		we go.1P.PL	on	sea,	if	wake-up.1P.PL	early.	
		‘We will go to the sea, if (we) wake up early’.						
	Unreal conditionals (4)	*esli by*	*sestra*	*pobedila*,	*papa*	*kupil*	*by*	*cvety.*
		if SUBJ	sister.NOM	won.FEM.SG	father	bought.MASC.SG	SUBJ	flowers.ACC
		‘We the sister had won, the father would have bought flowers’.						
	Subject relatives (8)	*jeto lošadka*,	*kotoraja*	*dognala*	*korovu.*			
		this horse.FEM.NOM	which.FEM.NOM	caught-up.FEM.SG	cow.FEM.ACC			
		‘This is a/the horse that caught-up-with a/the cow’.						
	Object relatives (8)	*jeto*	*medved’*,	*kotorogo*	*obmanula*	*lisa.*		
		this	bear.MASC.NOM	which.MASC.ACC	fooled.FEM.SG	fox.FEM.NOM		
		‘This is a/the bear a/the fox fooled’.						

**Table A2:** Structures tested in the Hebrew SRep task with examples (number of sentences per structure in brackets).

Level	Structure	Example						
1	SVO (8)	*hu axal.MASC.SG*	*tapuax axarey*	*ha-*	*ši’ur*			
		he ate	apple after	DEF-	lesson			
		‘He ate an apple after the lesson’.						
	Biclausal sentences with	*ha-banot*	*qonot*	*matanot ve*	*ha- banim*	*mesaxaqim*	*be-kadur*	
	coordination (4)	DEF-girls	buy.FEM.PL	presents and	DEF- boys	play.MASC.PL	in- ball.	
		‘The girls buy presents and the boys play with a ball’.						
	Biclausal sentences with	*ha- talmidot*	*ohavot*	*še-*	*ha- mora*	*me’axeret*		
	subordination (4)	DEF- pupils.FEM.PL	love.FEM.PL	that-	DEF- teacher	comes.late.FEM.SG	
		‘The pupils love that the teacher gets late’.					
2	Object questions (4)	*eyze*	*ganav*	*ha- shoteret*	*tafsa*	*ba-*	*layla*	
		which.MASC	thief	DEF- policewoman.FEM	caught.3P. FEM.SG	in+DEF	night	
		‘Which boy did the teacher frighten?’						
	Oblique questions (4)	*im*	*eyze*	*xaver*	*ha- yeled*	*rav*		
		with	which.MASC	friend.MASC	DEF- boy	quarrel.3P.MASC.SG		
		‘With which friend did the boy quarrel?’						
	Object relatives (4)	*zot*	*ha-*	*yalda*	*še*	*ha- iša*	*nishqa.*
		that	DEF-	girl.FEM.SG	that	DEF- woman.FEM.SG	kissed.3P. FEM.SG	
		‘I saw the dog that the horse pushed’.						
	Oblique relatives (4)	*ra’inu*	*et*	*ha-yeled*	*še ha- sus*	*ba’at*	*bo*	
		saw.1P.PL	ACC	DEF- boy.MASC.SG	that DEF- horse	kicked.3P.MASC.SG	in+him	
		‘(We) saw the boy that the horse kicked (into him)’.						
3	Real conditionals (4)	*im*	*yihye*	*xam nelex*	*lisxot*	*ba-*	*yam.*	
		if	will+be	hot will-go.1P.PL	to+swim	in+DEF	sea.	
		‘If it is hot, we will go to swim to the sea’.						
	Unreal conditionals (4)	*ha- shaxen*	*haya*	*qosher*	*et ha-kelev*	im hu haya nexmad.		
		DEF- neighbor.MASC.SG	was. MASC.SG	tie.MASC.SG	ACC DEF- dog	if he was nice.		
		‘The neighbor would have tied up the dog if he were nice’.						
	Biclausal sentences with	*kedey*	*le’efot*	*uga*	*qaninu*	*xalav*	*u- beycim.*	
	phrasal conjunctions (8)	in-order	to+bake	cake	bought.1P.PL	milk	and eggs.	
		‘In order to bake a cake, we bought milk and eggs’.						
	VSO (8)	*ba-*	*qayic*	*halxu*	*ha- yeladim*	*la- yam.*		
		in+DEF	summer	went.3P.PL	DEF- children.MASC.PL	to+DEF- *sea.*	
		‘In the morning the girl jumped on the rope’.						
